# Upregulation of SIRT6 predicts poor prognosis and promotes metastasis of non-small cell lung cancer via the ERK1/2/MMP9 pathway

**DOI:** 10.18632/oncotarget.9750

**Published:** 2016-05-31

**Authors:** Lihong Bai, Gengpeng Lin, Longhua Sun, Yangli Liu, Xinyan Huang, Chuangjie Cao, Yubiao Guo, Canmao Xie

**Affiliations:** ^1^ Respiratory Department, The First Affiliated Hospital of Sun Yat-Sen University, Institute of Respiratory Diseases of Sun Yat-Sen University, Guangzhou, Guangdong, People's Republic of China; ^2^ Respiratory Department, Nanchang Hospital of Integrative Traditional Chinese and Western Medicine, Jiangxi University of Traditional Chinese Medicine, Nanchang, Jiangxi, People's Republic of China; ^3^ Department of Pathology, The First Affiliated Hospital of Sun Yat-Sen University, Guangzhou, Guangdong, People's Republic of China

**Keywords:** non-small cell lung cancer, ERK1/2, MMP9, SIRT6, biomarker

## Abstract

Sirtuin6 (SIRT6), a member of the sirtuins protein family, plays multiple complex roles in cancer. Here, we report that elevated SIRT6 expression was correlated with clinicopathological parameters such as T and N classification in non-small cell lung cancer (NSCLC) patient tumors. SIRT6 overexpression in NSCLC cell lines increased extracellular signal-regulated kinase (p-ERK)1/2 phosphorylation, activated matrix metalloproteinase 9 (MMP9) and promoted tumor cell migration and invasion. Upon treatment with a specific mitogen-activated protein kinase (MEK) 1/2 inhibitor, these effects were abolished. Our results demonstrate SIRT6 upregulation in NSCLC for the first time and suggest a functional role for SIRT6 in promoting migration and invasion through ERK1/2/MMP9 signaling. SIRT6 may serve as a potential therapeutic target in NSCLC and its utility as a prognostic indicator warrants further study.

## INTRODUCTION

Lung cancer is the most commonly diagnosed cancer and is a leading cause of cancer-related morbidity worldwide, with 1.6 million new cases and 1.4 million deaths annually [1, 2]. Of the two major types of lung cancer, small cell lung carcinoma (SCLC) and non-small cell lung carcinoma (NSCLC), NSCLC accounts for 80–90% of cases and has a 5-year survival rate less than 15% [3, 4]. NSCLC is relatively refractory to both therapeutic modalities commonly used in lung cancer treatment, chemotherapy and radiation [5, 6]. Moreover, most patients are diagnosed with highly invasive, unresectable NSCLC, associated with poor outcome [7]. Metastasis resulting from later-stage disease is thought to be the major cause of death in lung cancer. Therefore, identification of novel targets to combat metastasis is critical and urgent.

Sirtuins (SIRTs) are a family of NAD^+^-dependent deacetylases that are highly conserved from lower organisms to humans. In mammals, seven different SIRTs (SIRT1–7) are linked to the regulation of critical biological processes, including metabolism, genomic stability, cell division, differentiation, survival, senescence and organismal lifespan [8, 9]. In addition, SIRT family members are thought to play roles in cancer development [10]. SIRT6 is located on a chromosomal locus (19p13.3) that is a frequent breakage site in human acute myeloid leukemia [11]. SIRT6 is overexpressed in several cancers, including prostate and endometrioid carcinomas, and keratinocyte-derived skin squamous cell carcinomas [12–14]. In contrast, SIRT6 is downregulated in pancreatic cancer, head and neck squamous cell carcinomas, human hepatocellular carcinoma (HCC) and colorectal carcinoma [15–17]. In human HCC, SIRT6 may act as a tumor suppressor, given that ectopic SIRT6 overexpression inhibits HCC cell growth [17, 18]. In breast cancer, high nuclear expression of SIRT6 is predictive of poor prognosis [8]. SIRT6 regulates Ca^2+^ responses to promote pancreatic cancer cell migration [15]. In contrast, many SIRT6 biological functions are associated with anti-cancer effects, including its role in cancer cell apoptosis, enhancing sensitivity to radiation damage and reducing cell viability [12]. Therefore, the role of SIRT6 in cancer is complex, with some studies supporting a tumor-suppressive role, and others a cancer-promoting role. In particular, the molecular mechanism(s) of SIRT6 activity in NSCLC are largely unknown.

In the present study, we found that SIRT6 is upregulated in NSCLC cell lines and patient-derived tumor tissues. SIRT6 overexpression was associated with clinicopathological features and prognosis in NSCLC. Silencing of endogenous SIRT6 reduced NSCLC cell migration and invasion, whereas ectopic SIRT6 overexpression promoted migration and invasion. Moreover, we demonstrated that SIRT6 promotes NSCLC cell invasion through the ERK1/2/MMP9 pathway.

## RESULTS

### SIRT6 is upregulated in NSCLC cell lines and tumor tissues

**Figure 1:**
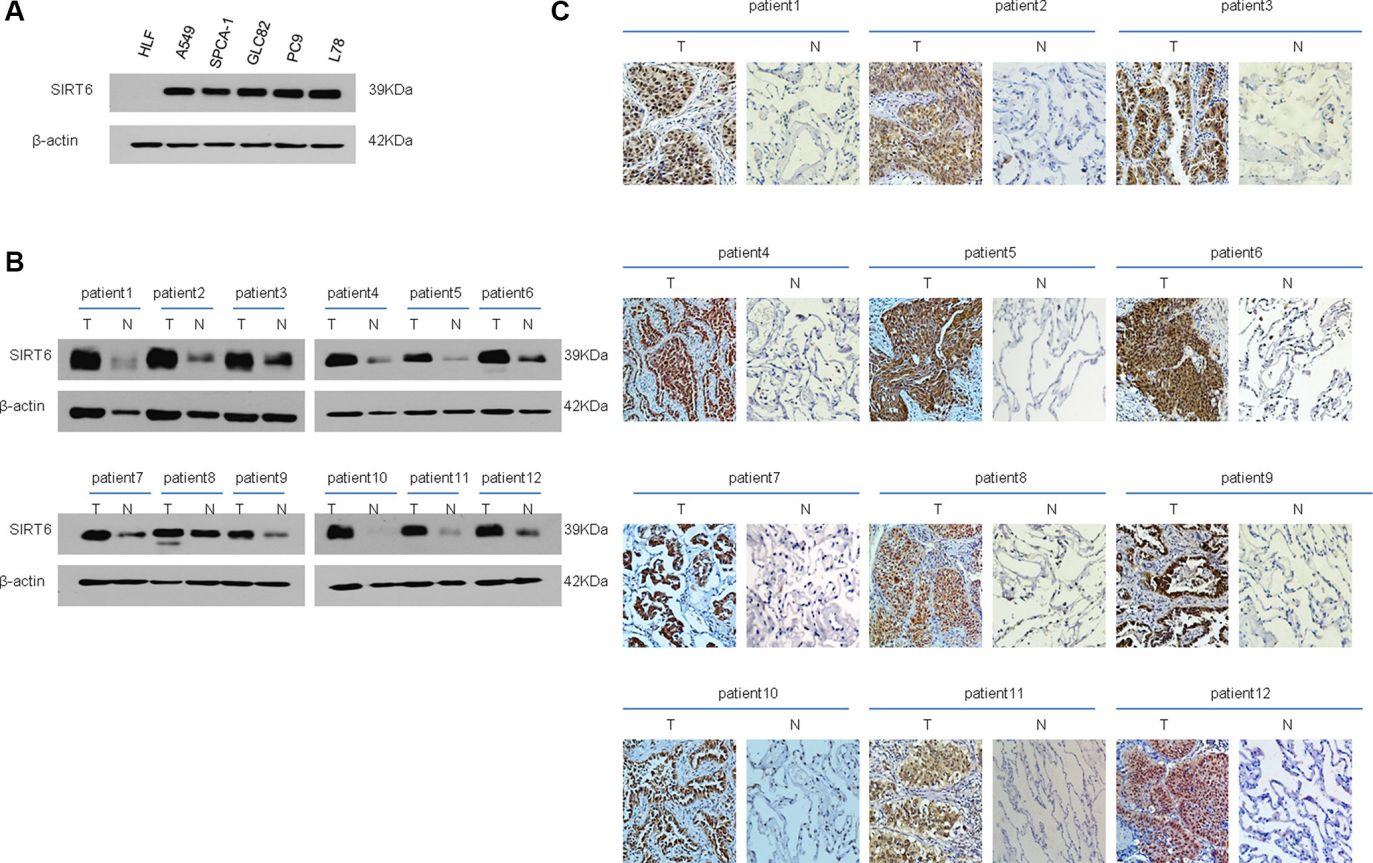
SIRT6 expression is elevated in both NSCLC cell lines and patient-derived cancer tissues Western blotting analysis of SIRT6 expression in NSCLC cell lines (**A**) Western blotting (**B**) and IHC (**C**) analysis of SIRT6 expression in primary NSCLC (T) and paired adjacent non-cancerous lung tissue samples (N). Protein levels were normalized with β-actin.

SIRT6 protein levels in both human NSCLC cell lines, including A549, SPC-A1, GLC82, PC9 and L78, and a human lung fibroblast (HLF) cell line, were assessed using western blotting analysis. All NSCLC cell lines expressed higher SIRT6 levels than did HLF cells (Figure 1A). Western blotting and immunohistochemical (IHC) staining demonstrated that SIRT6 was upregulated in 12 patient-derived NSCLC tissue samples as compared with paired adjacent noncancerous tissues (Figure 1B and 1C).

### Correlations between SIRT6 expression and NSCLC clinical features

We analyzed SIRT6 expression in 174 paraffin-embedded archived NSCLC tissue samples using IHC. Mean patient age was 59.3 years (range, 31–84 years), and the median follow-up period was 30 months (range, 0–120 months). A total of 128 deaths were reported during the follow-up period. SIRT6 was highly expressed in 128 of 174 (73.6%) human NSCLC samples. Spearman's correlation analysis indicated an association between high SIRT6 expression and clinical stage (*P* = 0.026), T classification (*P* = 0.016) and N classification (*P* = 0.019). However, SIRT6 overexpression was not associated with other clinicopathological parameters, including gender, age, M classification, histology subtypes and differentiation status (Table 1).

**Table 1:** Correlation between SIRT6 expression and clinicopathological features of NSCLC patients

Characteristics	Number of cases	Low expression	High expression	Chi-square test *P*-value	Fisher's exact test *P*-value
**Gender**	174				
Male	119	32	87	0.842	1.000
Female	55	14	41		
**Age**					
> 60 y	89	26	63	0.395	0.492
< = 60 y	85	20	65		
**Clinical stage**					
I + II	117	37	80	0.026	0.029
III + IV	57	9	48		
**T classification**					
T1 + T2	128	40	88	0.016	0.019
T3 + T4	46	6	40		
**N classification**					
N0 + N1	138	42	96	0.019	0.020
N2 + N3	36	4	32		
**M classification**					
M0	157	44	113	0.149	0.245
M1	17	2	15		
**Histological differentiation**					
Poor	77	19	58	0.827	0.881
Moderate	88	25	63		
Well	9	2	7		
**Tissue subtypes**		35	109		
Adenocarcinoma	144	10	18	0.342	0.249
Squamous cell cancer	28				
others	2	1	1		

### SIRT6 prognostic significance in NSCLC Patients

**Figure 2:**
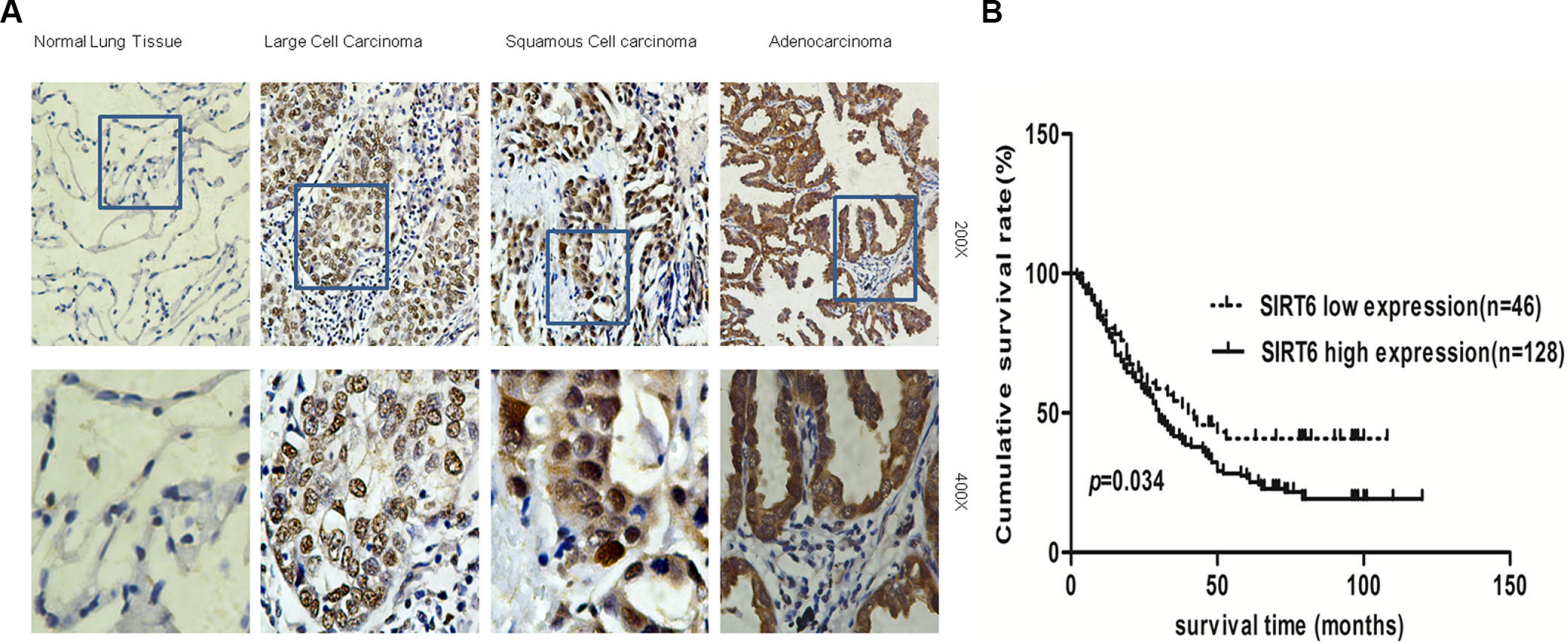
SIRT6 is overexpressed in NSCLC Representative images of SIRT6 IHC analysis in normal lung tissues and different NSCLC subtypes (**A**) Survival curves of high and low SIRT6-expressing in NSCLC patients (total *n* = 174; *P* = 0.034) (**B**).

Statistical analyses showed that high SIRT6-expressing NSCLC patients had a lower cumulative survival rate as compared with low SIRT6-expression patients (*P* = 0.034; Figure 2B). The cumulative 5-year survival rate was 53.1% (95% confidence interval [CI]: 44.48–61.72%) for patients with low SIRT6 expression and was 40.8% (95% CI: 26.49–55.11%) for patients with high expression.

### SIRT6 promotes NSCLC cell migration and invasion

**Figure 3:**
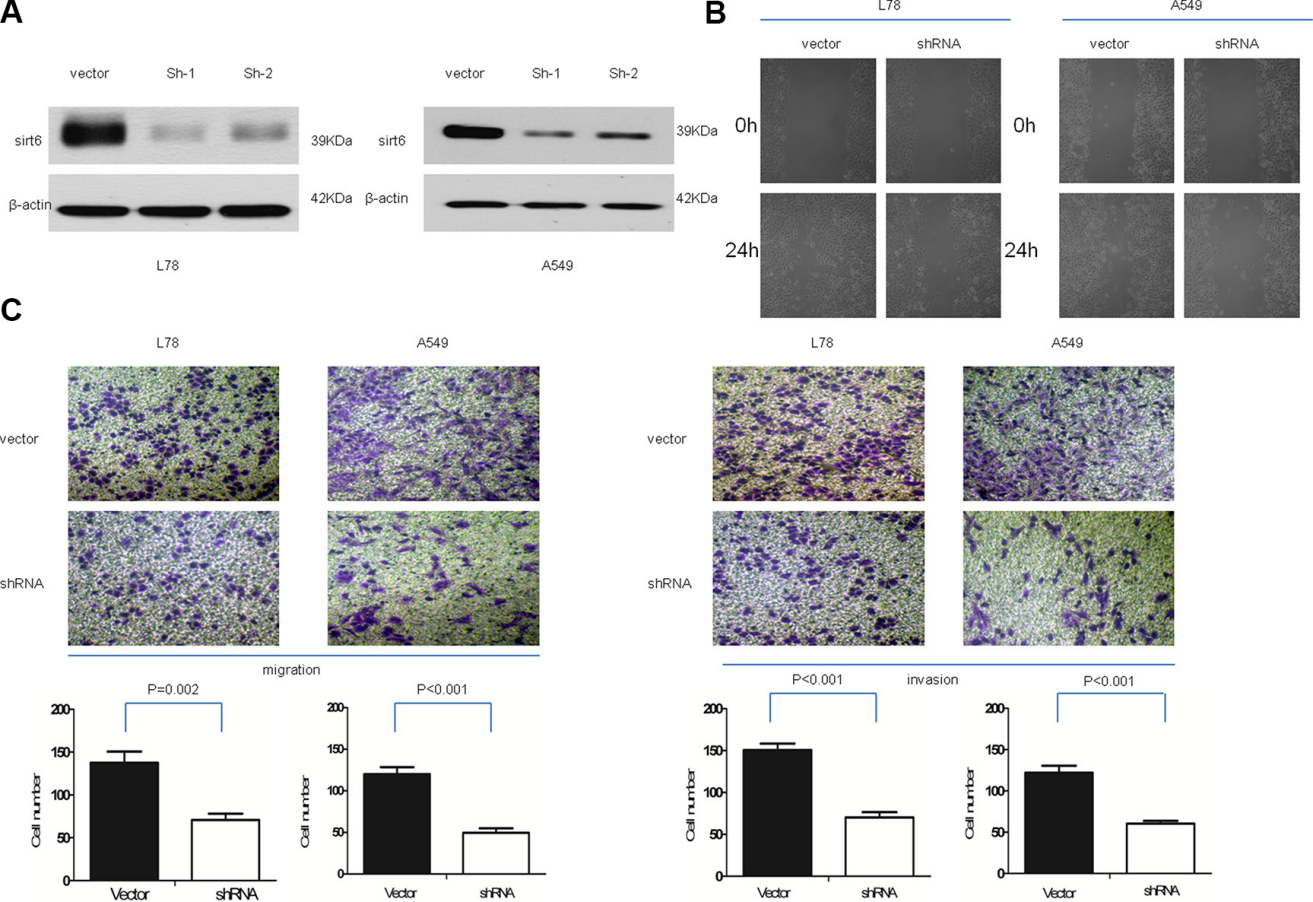
SIRT6 knockdown decreases NSCLC cell migration and invasion SIRT6 western blotting analysis (**A**) and wound healing assays (**B**) in vector-control cells (vector) and SIRT6-shRNA-transduced NSCLC cells (sh1 and sh2); β-actin was used as the loading control for western blotting. Representative micrographs and cell migration and invasion quantification from the transwell migration assay, with and without Matrigel (**C**) Images represent data from three independent trials with two technical replicates per trial.

**Figure 4:**
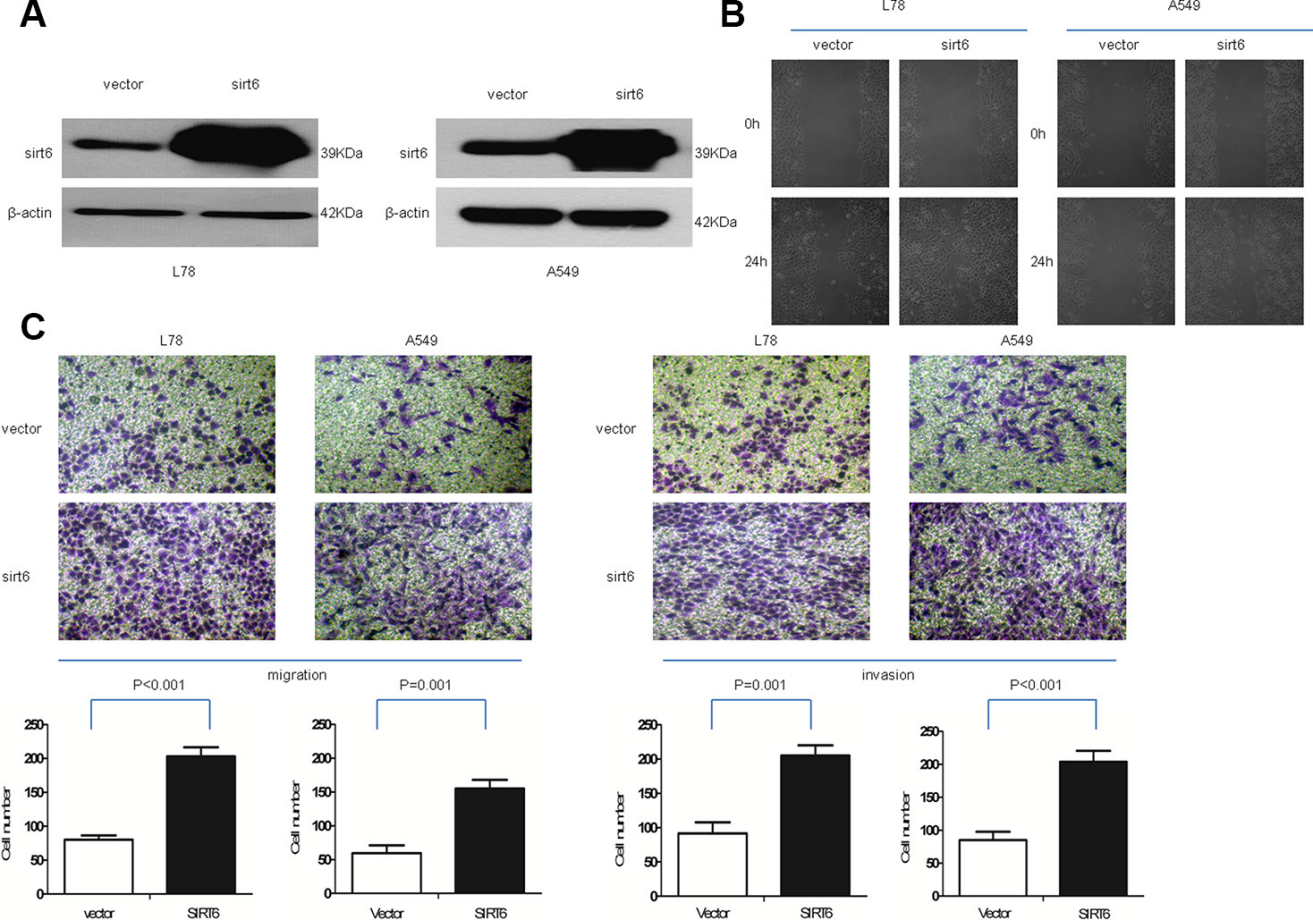
Ectopic SIRT6 expression enhances NSCLC cell migration and invasion SIRT6 western blotting analysis (**A**) and wound healing assays (**B**) in A549-vector (vector), A549-SIRT6 (SIRT6), L78-vector (vector) and L78-SIRT6 (SIRT6) cells; β-actin was used as the loading control for western blotting. Representative micrographs and cell migration and invasion quantification from the transwell migration assay, with and without Matrigel (**C**) Images represent data from three independent trials with two technical replicates per trial.

We investigated the effects of SIRT6 overexpression on NSCLC cell invasion. NSCLC cells were engineered to stably overexpress or silence SIRT6 (Figures 3A and 4A). Wound-healing assays showed that ectopic SIRT6 expression accelerated NSCLC cell migration (Figure 4B). Transwell assays (with or without Matrigel) revealed that SIRT6 overexpression increased the migration and invasion rates of A549 and L78 cells (Figure 4C), whereas SIRT6 silencing reduced migration and invasion (Figure 3C).

### SIRT6 promotes migration and invasion via ERK1/2/MMP9

**Figure 5:**
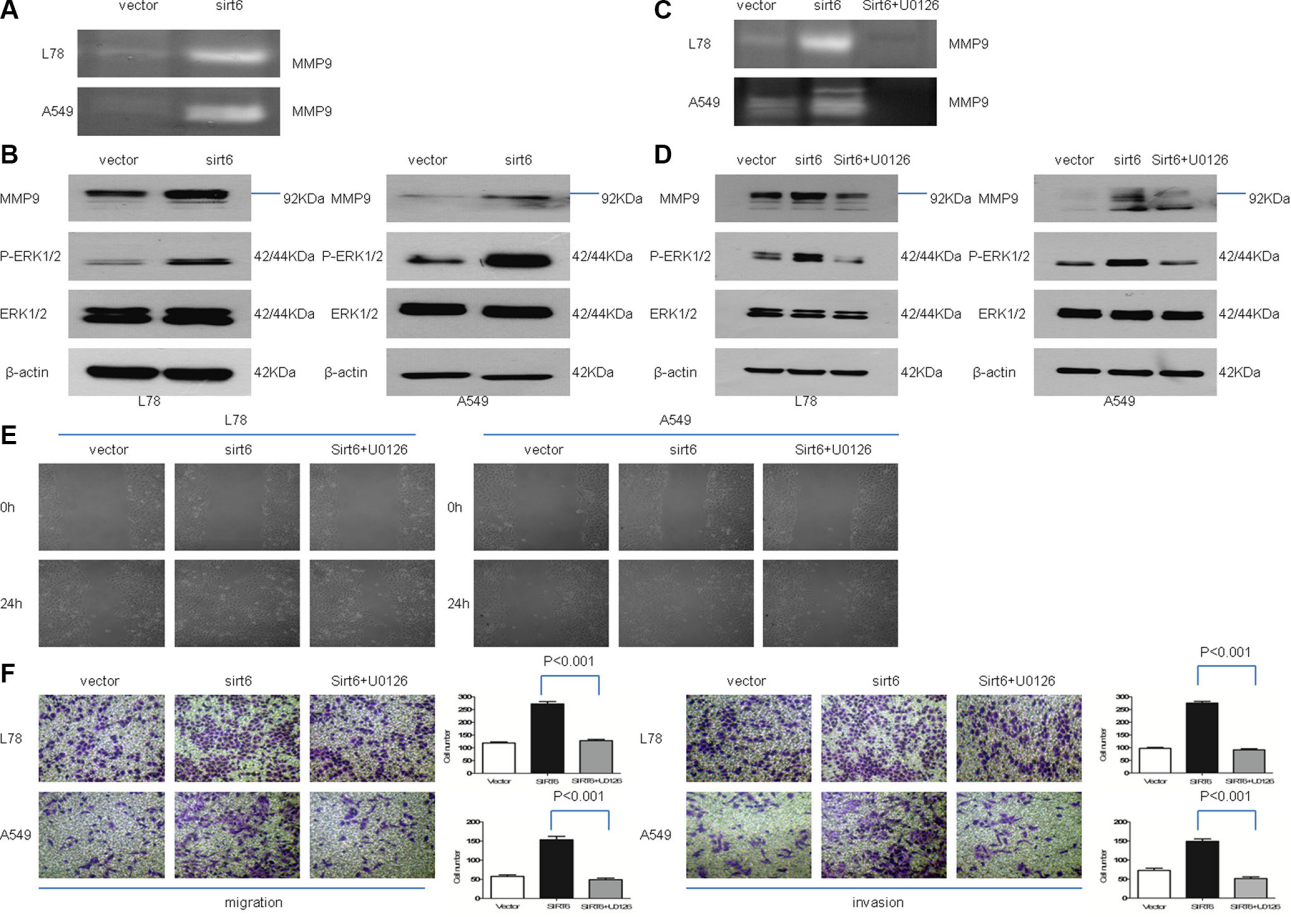
SIRT6 promotes NSCLC cell migration and invasion through ERK1/2//MMP9 Zymographic analysis of MMP9 activity in conditioned medium from SIRT6-overexpressing NSCLC cells and corresponding vector control cells (**A**) Western blotting analysis of p-ERK1/2, ERK1/2 and MMP9 in indicated cells (**B** and **D**) SIRT6 overexpression increased p-ERK1/2 and MMP9 expression, and treatment with the specific MEK1/2 inhibitor U0126 abolishes these effects. Zymographic analysis of MMP9 activity in conditioned medium from SIRT6-overexpressing cells, with or without U0126 (**C**) Wound healing assay results showed that stable SIRT6 overexpression promotes cell migration, which is abolished by concomitant treatment with U0126 (**E**) Migration and invasion assays using a transwell assay system (**F**) SIRT6-overexpressing cells were treated with U0126 or vehicle alone (without U0126). Representative images and quantification of migration and invasion are shown.

We examined the effects of SIRT6 expression on the ERK1/2/MMP9 pathway, which is involved in lung cancer metastasis and invasion. ERK is a member of the mitogen-activated protein kinase (MAPK) signaling pathway, which positively regulates activator protein 1 (AP-1). AP-1 acts as a master regulator of tumor cell migration and invasion by targeting genes such as MMP9. In A549 and L78 cells stably overexpressing SIRT6, MMP9 levels and activity and ERK1/2 phosphorylation were elevated compared to control cells (Figure 5A, 5B and 5D). Treatment of cells with the specific MEK1/2 inhibitor U0126 abrogated SIRT6 overexpression-mediated invasion and migration and MMP9 expression/activity (Figure 5C–5F). These results demonstrated that SIRT6 promotes invasion and migration through the ERK1/2/MMP9 pathway.

## DISCUSSION

To the best of our knowledge, this is the first report correlating SIRT6 overexpression with clinicopathologic NSCLC characteristics, such as tumor stage. In addition, we found that SIRT6 overexpression predicts poor NSCLC patient prognosis. SIRT6 is highly expressed in thymus, skeletal, brain and muscle tissues [24, 25]. SIRT6 has two major biochemical activities, functioning as (1) a deacetylase and (2) a mono-ADP ribosyltransferase [26, 27]. SIRT6 participates in numerous biological processes, including maintaining genomic stability, modulating senescence and development of age-related diseases [8, 9]. Recently, SIRT6 was implicated in cancers [10], although the role of SIRT6 in various cell types is different. In human HCC, SIRT6 inhibits survivin to control cancer initiation via an AP-1-dependent regulatory network [28]. SIRT6 overexpression induced apoptosis in HT1080 (fibrosarcoma cells), MEF (mouse embryonic fibroblasts), HeLa (human cervical cancer cells) and HCA2 (human sigmoid colon carcinoma) cells, but not in their non-cancerous/normal counterparts [29]. These data support a tumor suppressor role for SIRT6 in cancer.

On the other hand, SIRT6 inhibition reduced prostate cancer cell viability and increased apoptosis, suggesting that SIRT6 may promote tumorigenesis [12]. SIRT6 promoted pancreatic cancer cell migration by inducing cytokines such as interleukin-8 and tumor necrosis factor in a Ca^2+^-dependent manner [15]. There is thus evidence to support conflicting functions for SIRT6 in cancer, either as a tumor suppressor and as a cancer-promoting factor under different circumstances. However, the role of SIRT6 in NSCLC is debated in the literature. Han, et al. [30] showed that SIRT6 suppresses NSCLC cell proliferation through Twist1 inhibition. Cai, et al. [31] showed that SIRT6 overexpression can reduce cell proliferation, change the cell distribution of the cell cycle and induce apoptosis via Bcl-2 downregulation and Bax and cleaved caspase-3 upregulation. However, YOKO, et al. [32] reported that SIRT6 knockdown did not affect A549 cell proliferation or Bcl-2 expression in this cell line. Similarly, Kim, et al. [33] found that cAMP reduces SIRT6 expression to enhance apoptosis via inhibition of the Raf-MEK-ERK pathway.

In this study, we found a positive correlation between SIRT6 overexpression and TNM stage N classification in NSCLC. Our results also showed that stable SIRT6 knockdown in NSCLC cells reduced migration and invasion, whereas ectopic SIRT6 overexpression increased migration and invasion. We speculate that SIRT6 promotes NSCLC metastasis. Consistent with this hypothesis, a previous study suggested that elevated SIRT6 promotes pancreatic cancer cell migration and invasion and may play a vital role in disease progression [15].

Tumor metastasis is a multistep process, and considerable evidence shows that MMPs drive metastasis by degrading the extracellular matrix (ECM) [34]. Type I and IV collagens are the major ECM components. Under certain conditions, MMP9 degrades these collagens, thereby expediting and facilitating cancer cell invasion and metastasis [7, 35]. MMP9 has been found in various cancer types, including glioma, lung cancer, pancreatic cancer and osteosarcoma [34]. MMP9 upregulation was predictive of poor prognosis in patients with lung cancer, glioma or colorectal cancer [43]. The MMP9 gene promoter regions contain cis-elements for the Sp1 transcription factor, and ERK activation is crucial for Sp1-mediated MMP9 expression [36, 37]. MMP9 reportedly also contains a highly conserved proximal AP-1 binding site. ERK belongs to the MAPK family of kinases, which transduce a wide variety of extracellular stimuli into intracellular cascades and regulate a number of transcription factors, including AP-1 [38, 39]. MAPKs also participate in many cancer processes, such as cell proliferation, angiogenesis, migration and invasion [34, 40]. In addition, the ERK1/2/MMP9 pathway also reportedly modulates migration and invasion in colorectal cancer, prostate cancer and NSCLC by targeting various genes [41–44]. In this study, we demonstrated a link between SIRT6 overexpression and increased ERK activation, as indicated by increased ERK1/2 phosphorylation without changes in total ERK1/2 levels. We observed subsequent MMP9 upregulation, ultimately leading to enhanced NSCLC cell migration and invasion. These SIRT6-mediated effects were MEK1/2-dependent, since concomitant treatment with a MEK1/2 inhibitor abolished the above effects. This report demonstrates SIRT6 upregulation in NSCLC for the first time, and suggests a functional role for SIRT6 in promoting migration and invasion through ERK1/2/MMP9 signaling.

In conclusion, our study demonstrated that SIRT6 upregulation was associated with an invasive NSCLC phenotype in patients and may promote NSCLC development and progression. Furthermore, we demonstrated that SIRT6 promoted metastasis through the ERK1/2/MMP9 pathway. SIRT6 may serve as a potential therapeutic target in NSCLC and its utility as a prognostic indicator warrants further study.

## MATERIALS AND METHODS

### Cell lines and cultures

The A549, SPC-A1, GLC-82 and PC-9 human lung adenocarcinoma cell lines, the L78 human squamous lung cancer cell line and the HLF human lung fibroblast cell line were used in this study. A549, PC9 and HLF cells were obtained from Cell Bank, Chinese Academy of Sciences (Shanghai, China), and were maintained in our laboratory. SPC-A1, GLC82 and L78 cells were kind gifts from Prof. Liantang Wang at the Department of Pathology in the First Affiliated Hospital of Sun Yat-Sen University. Cells were grown in Roswell Park Memorial Institute (RPMI) 1640 medium (Gibco, BRL, Carlsbad, CA) supplemented with 10% fetal bovine serum (FBS; HyClone, Logan, UT) at 37°C in a humidified incubator with 5% CO_2_.

### Plasmid construction and retroviral infection

The pBaBb-puromycin plasmid was used to generate pBaBb-puromycin plasmid-SIRT6, the SIRT6 expression vector. The pSUPER-retro-puro vector was used to generate pSUPER-SIRT6-ShRNA, the plasmid expressing SIRT6-specific shRNA. A549 and L78 cells were infected with the retrovirus expressing SIRT6 or pBaBb-puromycin plasmid alone (empty vector). SIRT6 was also knocked down in A549 and L78 cells by transduction with control or SIRT6-specific shRNA-harboring retroviruses. Stable cell lines overexpressing SIRT6 or silenced for SIRT6, and the corresponding control cell lines, were selected with puromycin for 10–14 days beginning 48 h after infection. Cell lysates prepared in the sampling buffer were resolved by SDS-PAGE and SIRT6 levels were assessed by western blotting. Ectopic SIRT6 coding sequence was amplified by polymerase chain reaction (PCR). The primer sequences were: forward: 5′-TTCTTCGAAATGTCGGTGAATTACGCGGC-3′; reverse: 5′-CTAGCTAGCTCAGCTGGGGACCGCCTT GG-3′. The following *SIRT6* sequences were targeted by shRNA: SIRT6-SH1, CCGGGAAGAATGTGCCAA GTGTAAGCTCGAGCTTACACTTGGCACATTCTTCT TTTTG and SIRT6-SH2, CCGGCAAGTTCGACACCA CCTTTGACTCGAGTCAAAGGTGGTGTCGAACTT GTTTTTG.

### Patients and tissue specimens

Fresh tumor tissue samples, along with paired non-cancerous lung tissue samples, were obtained during surgery from 12 NSCLC patients treated at the First Affiliated Hospital of Sun Yat-sen. Paraffin-embedded, archived NSCLC samples were obtained from 174 patients diagnosed with NSCLC between January 2004 and December 2009 at the Department of Pathology in the First Affiliated Hospital of Sun Yat-sen. Histologic characterization and clinicopathological staging of the samples were determined according to WHO criteria [19] and current Union for International Cancer Control tumor–node–metastasis (TNM) classification [20–21]. Patient clinical information is summarized in Table 1. Pertinent follow-up information was available for all patients. Written informed patient consent and study approval from the Institutional Research Ethics Committee were obtained.

### Western blotting analysis

Western blotting was performed as previously described [22, 23]. Briefly, cells were lysed in radio-immunoprecipitation buffer, and clarified lysates were resolved by SDS-PAGE and transferred onto polyvinylidene fluoride membranes (Millipore, Bedford, MA). Antibodies specific for SIRT6 (Abcam, 1:4000), ERK1/2 (Cell Signaling Technology, Boston, MA), p-ERK1/2 (Cell Signaling Technology) and MMP9 (Cell Signaling Technology, Danvers, MA) were used for western blotting analysis with β-actin (Cell Signaling Technology, Danvers, MA, USA) as the internal loading control. Signals were detected using enhanced chemiluminescence-based methods.

### Immunohistochemistry

IHC analysis was performed to study altered protein expression in 174 human NSCLC tissues using previously described methods [22]. Slides (paraffin-embedded sections) were incubated with polyclonal rabbit anti-human SIRT6 (Abcam, 1:400) overnight at 4°C. Immunohistostaining was scored separately by two independent investigators (Ran Wang and Minghui Zhang) blinded to histopathological features and patient data, and the average of these two scores was calculated for each sample. Scores were determined by assessing both staining intensity and the proportion of positively stained tumor cells. The proportion of positively stained tumor cells was graded as follows: 1, ≤ 25% positive tumor cells; 2, > 25% to ≤ 50% positive tumor cells; 3, > 50% to ≤ %75% positive tumor cells; and 4, > 75% positive tumor cells. Staining intensity was recorded on a scale of 0–3 with: 0, no staining, negative; 1, weak staining, light yellow; 2, moderate staining, yellowish brown; and 3, strong staining, brown. SIRT6 staining index (SI) was calculated (values 0–12) as follows: SI = staining intensity × proportion of positively stained tumor cells. An SI score > 6 was used to define tumors with high SIRT6 expression and ≤ 6 represented tumors with low expression.

### Wound-healing assay

A549 and L78 cells were grown to confluence in cell culture dishes. A wound was inflicted on the monolayer using a 100 μL pipette tip. Cells were maintained in serum-free medium and allowed to migrate for 24 h before images of cells that had migrated into the wound area were taken.

### Migration and invasion assays

Migration and invasion assays were performed as described previously [22]. Briefly, cells were plated onto cell culture inserts with 8μm microporous filters (Corning) coated with (invasion) or without (migration) 40 μL Matrigel (1:8 dilution; BD Biosciences, Bedford, MA) and incubated for 24 h. Cells in the upper filters (inside the inserts) were removed, and cells that had migrated into or invaded the lower filters were fixed in 4% paraformaldehyde, stained with crystal violet and counted under a microscope. The number of migrated or invaded cells was counted in five random optical fields for each filter (100 × magnification). For A549 and L78 cells, 2 × 10^4^ and 4 × 10^4^ cells were plated onto each insert, respectively. To test the effect of the specific MEK1/2 inhibitor U0126 (Sigma) on the migratory and invasive abilities of cells, A549 and L78 cells were pretreated with 10 μM U0126 for 30 min before migration and invasion assays were performed. Experiments were performed in triplicate.

### Zymography

Cells plated in 6-well plates were cultured in fresh RPMI 1640 medium for another 48 h after a particular treatment. Culture medium was then collected and centrifuged, and the supernatant was preserved. Supernatants were concentrated using ultrafiltration (Millipore, Billerica, MA) and protein concentration was measured using the BCA kit (CWBiotech, Beijing, China). Protein (40 μg) was loaded and separated on a 10% SDS polyacrylamide gel containing 1% gelatin by electrophoresis at 27mA/gel and 4°C. Gels were then processed according to the gelatin zymography kit instructions (Applygen, Beijing, China).

### Statistical analysis

Data are expressed as mean ± standard deviation (SD) of values from three independent trials. Groups were compared using Student^'^s *t*-test or one-way analysis of variance (ANOVA). The χ^2^ test and Spearman's correlation analysis were used to analyze relationships between SIRT6 expression and clinicopathological characteristics. The Kaplan-Meier method was used to plot survival curves and the log-rank test was used to compare survival curves. *P* < 0.05 was considered statistically significant. Statistical analyses were performed using SPSS 13.0 software.

## References

[1] Ferlay J, Shin HR, Bray F, Forman D, Mathers C, Parkin DM (2010). Estimates of worldwide burden of cancer in 2008: GLOBOCAN 2008. Int J Cancer.

[2] Jemal A, Siegel R, Xu J, Ward E (2010). Cancer statistics, 2010. CA Cancer J Clin.

[3] Jemal A, Siegel R, Ward E, Murray T, Xu J, Thun MJ (2007). CA Cancer J Clin.

[4] Kwon MJ, Seo J, Kim YJ, Kwon MJ, Choi JY, Kim TE, Lee DH, Park S, Shin YK, Han J, Choi YL (2013). Prognostic significance of CD151 overexpression in non-small cell lung cancer. Lung Cancer.

[5] Pei J, Lou Y, Zhong R, Han B (2014). Mmp9 activation triggered by epidermal growth factor induced foxo1 nuclear exclusion in non-small cell lung cancer. Tumour Biol.

[6] Kuwabara K, Matsuda S, Fushimi K, Anan M, Ishikawa KB, Horiguchi H, Hayashida K, Fujimori K (2009). Differences in practice patterns and costs between small cell and non-small -cell lung cancer patients in Japan. Tohoku J Exp Med.

[7] Jian H, Zhao Y, Liu B, Lu S (2014). SEMA4b inhibits MMP9 to prevent metastasis of non-small cell lung cancer. Tumour Biol.

[8] Khongkow M, Olmos Y, Gong C, Gomes AR, Monteiro LJ, Yagüe E, Cavaco TB, Khongkow P, Man EP, Laohasinnarong S, Koo CY, Harada-Shoji N, Tsang JW (2013). SIRT6 modulates paclitaxel and epirubicin resistance and survival in breast cancer. Carcinogenesis.

[9] Grimley R, Polyakova O, Vamathevan J, McKenary J, Hayes B, Patel C, Smith J, Bridges A, Fosberry A, Bhardwaja A, Mouzon B, Chung CW, Barrett N (2012). Over expression of wild type or a catalytically dead mutant of Sirtuin 6 does not influence NF-kappaB responses. PLOS One.

[10] Bosch-Presegué L, Vaquero A (2011). The dual role of sirtuins in cancer. Genes Cancer.

[11] Mahlknecht U, Ho AD, Voelter-Mahlknecht S (2006). Voelter-Mahlknecht, Chromosomal organization and fluorescence *in situ* hybridization of the human Sirtuin 6 gene. Int J Oncol.

[12] Liu Y, Xie QR, Wang B, Shao J, Zhang T, Liu T, Huang G, Xia W (2013). Inhibition of SIRT6 in prostate cancer reduces cell viability and increases sensitivity to chemotherapeutics. Protein Cell.

[13] Lefort K, Brooks Y, Ostano P, Cario-André M, Calpini V, Guinea-Viniegra J, Albinger-Hegyi A, Hoetzenecker W, Kolfschoten I, Wagner EF, Werner S, Dotto GP (2013). A miR-34a-SIRT6 axis in the squamous cell differentiation network. EMBO J.

[14] Colas E, Perez C, Cabrera S, Pedrola N, Monge M, Castellvi J, Eyzaguirre F, Gregorio J, Ruiz A, Llaurado M, Rigau M, Garcia M, Ertekin T (2011). Molecular markers of endometrial carcinoma detected in uterine aspirates. Int J Cancer.

[15] Bauer I, Grozio A, Lasigliè D, Basile G, Sturla L, Magnone M, Sociali G, Soncini D, Caffa I, Poggi A, Zoppoli G, Cea M, Feldmann G (2012). The NAD+-dependent histone deacetylase SIRT6 promotes cytokine production and migration in pancreatic cancer cells by regulating Ca2+ responses. J Biol Chem.

[16] Lai CC, Lin PM, Lin SF, Hsu CH, Lin HC, Hu ML, Hsu CM, Yang MY (2013). Altered expression of SIRT gene family in head and neck squamous cell carcinoma. Tumour Biol.

[17] Sebastián C, Zwaans BM, Silberman DM, Gymrek M, Goren A, Zhong L, Ram O, Truelove J, Guimaraes AR, Toiber D, Cosentino C, Greenson JK, MacDonald AI (2012). The histone deacetylase SIRT6 is a tumor suppressor that controls cancer metabolism. Cell.

[18] Zhang ZG, Qin CY (2014). Sirt6 suppresses hepatocellular carcinoma cell growth via inhibiting the extracellular signal regulated kinase signaling pathway. Mol Med Rep.

[19] Jacques J, Hill DP, Shier KJ, Jindani A, Miller AB (1980). Appraisal of the World Health Organization classification of lung tumors. Can Med Assoc J.

[20] Mountain C.F (1997). Revisions in the International System for Staging Lung Cancer. Chest.

[21] Detterbeck FC, Boffa DJ, Tanoue LT (2009). The new lung cancer staging system. Chest.

[22] Lin G, Sun L, Wang R, Guo Y, Xie C (2014). Overexpression of muscarinic receptor 3 promotes metastasis and predicts poor prognosis in non-small-cell lung cancer. J Thorac Oncol.

[23] Sun L, Bai L, Lin G, Wang R, Liu Y, Cai J, Guo Y, Zhu Z, Xie C (2015). CUEDC2 down-regulation is associated with tumor growth and poor prognosis in lung adenocarcinoma. Oncotarget.

[24] Liszt G, Ford E, Kurtev M, Guarente L (2005). Mouse Sir2 homolog SIRT6 is a nuclear ADP-ribosyltransferase. J Biol Chem.

[25] Michishita E, Park JY, Burneskis JM, Barrett JC, Horikawa I (2005). Evolutionarily conserved and nonconserved cellular localizations and functions of human SIRT proteins. Mol Biol Cell.

[26] Michishita E, McCord RA, Berber E, Kioi M, Padilla-Nash H, Damian M, Cheung P, Kusumoto R, Kawahara TL, Barrett JC, Chang HY, Bohr VA, Ried T (2008). SIRT6 is a histone H3 lysine 9 deacetylase that modulates telomeric chromatin. Nature.

[27] Polyakova O, Borman S, Grimley R, Vamathevan J, Hayes B, Solari R (2012). Identification of novel interacting partners of Sirtuin6. PLOS One.

[28] Min L, Ji Y, Bakiri L, Qiu Z, Cen J, Chen X, Chen L, Scheuch H, Zheng H, Qin L, Zatloukal K, Hui L, Wagner EF (2012). Liver cancer initiation is controlled by AP-1 through SIRT6-dependent inhibition of survivin. Nat Cell Biol.

[29] Van Meter M, Mao Z, Gorbunova V, Seluanov A (2011). SIRT6 overexpression induces massive apoptosis in cancer cells but not in normal cells. Cell Cycle.

[30] Han Z, Liu L, Liu Y, Li S (2014). Int J Clin Exp Pathol.

[31] Cai Y, Sheng ZY, Liang SX (2014). Asian Pac J Cancer Prev.

[32] Azuma Y, Yokobori T, Mogi A, Altan B, Yajima T, Kosaka T, Onozato R, Yamaki E, Asao T, Nishiyama M, Kuwano H (2015). J Surg Oncol.

[33] Kim EJ, Juhnn YS (2015). J Biol Chem.

[34] Zhou B, Wu Q, Chen G, Zhang TP, Zhao YP (2012). NOP14 promotes proliferation and metastasis of pancreatic cancer cells. Cancer Lett.

[35] Poudel B, Kim DK, Ki HH, Kwon YB, Lee YM, Kim DK (2014). Downregulation of ERK signaling impairs U2OS osteosarcoma cell migration in collagen matrix by suppressing MMP9 production. Oncol Lett.

[36] Kuo L, Chang HC, Leu TH, Maa MC, Hung WC (2006). Src oncogene activates MMP-2 expression via the ERK/Sp1 pathway. J Cell Physiol.

[37] Liu WH, Chen YJ, Chien JH, Chang LS (2014). Amsacrine suppresses matrix metalloproteinase-2 (MMP-2)/MMP-9 expression in human leukemia cells. J Cell Physiol.

[38] Tang ZP, Cui QZ, Dong QZ, Xu K, Wang EH (2013). Ataxia-telangiectasia group D complementing gene (ATDC) upregulates matrix metalloproteinase 9 (MMP-9) to promote lung cancer cell invasion by activating ERK and JNK pathways. Tumour Biol.

[39] Risolino M, Mandia N, Iavarone F, Dardaei L, Longobardi E, Fernandez S, Talotta F, Bianchi F, Pisati F, Spaggiari L, Harter PN, Mittelbronn M, Schulte D (2014). Transcription factor PREP1 induces EMT and metastasis by controlling the TGF-beta-SMAD3 pathway in non-small cell lung adenocarcinoma. Proc Natl Acad Sci U S A.

[40] Xu L, Zhang Y, Wang H, Zhang G, Ding Y, Zhao L (2014). Tumor suppressor miR-1 restrains epithelial-mesenchymal transition and metastasis of colorectal carcinoma via the MAPK and PI3K/AKT pathway. J Transl Med.

[41] Kim HC, Kim YS, Oh HW, Kim K, Oh SS, Kim JT, Kim BY, Lee SJ, Choe YK, Kim DH, Kim SH, Chae SW, Kim KD (2014). Collagen triple helix repeat containing 1 (CTHRC1) acts via ERK-dependent induction of MMP9 to promote invasion of colorectal cancer cells. Oncotarget.

[42] Kato T, Fujita Y, Nakane K, Mizutani K, Terazawa R, Ehara H, Kanimoto Y, Kojima T, Nozawa Y, Deguchi T, Ito M (2013). CCR1/CCL5 interaction promotes invasion of taxane-resistant PC3 prostate cancer cells by increasing secretion of MMPs 2/9 and by activating ERK and Rac signaling. Cytokine.

[43] Lin F, Chengyao X, Qingchang L, Qianze D, Enhua W, Yan W (2015). CRKL promotes lung cancer cell invasion through ERK-MMP9 pathway. Mol Carcinog.

[44] Bjørnland K, Flatmark K, Pettersen S, Aaasen AO, Fodstad O, Maelandsmo GM (2005). Matrix metalloproteinases participate in osteosarcoma invasion. J Surg Res.

